# Translation and Validation of the Malayalam Version of the Subjective Happiness Scale

**DOI:** 10.1007/s11205-024-03448-y

**Published:** 2024-10-22

**Authors:** Kelly Cotton, Sanish Sathyan, Soumya Jacob, K. S. Shaji, Emmeline Ayers, Dristi Adhikari, Alben Sigamani, VG Pradeep Kumar, Joe Verghese

**Affiliations:** 1 Department of Neurology, Albert Einstein College of Medicine, 1225 Morris Park Avenue, Bronx, NY 10461 USA; 2 Christ University, Bengaluru, India; 3 Kerala University of Health Sciences, Thrissur, Kerala India; 4 Carmel Research Consultancy Pvt Ltd, Bengaluru, India; 5 Institute of Neurosciences, Baby Memorial Hospital, Kozhikode, India; 6 Department of Medicine, Albert Einstein College of Medicine, Bronx, NY USA

**Keywords:** Subjective happiness scale, Malayalam, Measurement, Validity, Well-being

## Abstract

The subjective happiness scale (SHS) is a brief instrument used to measure global subjective happiness that has been translated from its original English to many other languages. To date, there is no reported translation of this scale into Malayalam, a language spoken by over 32 million people especially in the southern state of Kerala, India. In the present study, 656 community-dwelling older adults participating in the Kerala Einstein study (KES) completed the Malayalam version of the SHS. The Malayalam version demonstrated high internal consistency and good convergent validity, as assessed by comparison to measures of depression and anxiety. We also used factor analysis to determine that the Malayalam version of the SHS has a unidimensional structure, akin to the original English as well as other language adaptations. Our study adds to the repertoire of tools to measure happiness in non-English-speaking populations, enabling future research to explore the foundations of well-being across diverse cultures.

**Supplementary Information:**

The online version contains supplementary material available at 10.1007/s11205-024-03448-y.

## Introduction

Over the past several decades, investigating happiness, one aspect of the broader “subjective well-being” (Diener, 1984), has been an increasingly prominent area of research. This includes examining who is happy (Diener et al., 1999; Myers & Diener, 1995), as well as where (Diener, 2000; Oishi et al., 2013), when (Oishi et al., 2013), and why (Diener et al., 2018; Lyubomirsky et al., 2005) people are happy. To better understand happiness and its determinants, it is first necessary to develop ways to measure this complex concept.

Happiness consists of both emotional (experiencing frequent positive feelings and less frequent negative feelings) and cognitive (evaluating one’s general satisfaction with life) components (Diener, 1984; Myers & Diener, 1995). As such, happiness is not simply the sum of the “good” things that happen, but instead reflects a balance of overall positive and negative feelings, and, importantly, one’s own subjective experience of life. The Subjective Happiness Scale (SHS) was developed to capture this multidimensional construct (Lyubomirsky & Lepper, 1999). The original version of the SHS demonstrated high levels of internal consistency, reliability, and construct validity across several different participant samples in both English and Russian. The SHS is short, consisting of only four items, and has since been used widely in English-speaking samples and translated into numerous languages, including, but not limited to, Spanish (Extremera & Fernández-Berrocal, 2014), German (Swami et al., 2009), Turkish (Dogan & Totan, 2013), Greek (Karakasidou et al., 2016), Chinese (Chien et al., 2020), and Tagalog (Swami et al., 2009), illustrating the practical utility of this instrument.

Recently, India became the most populous country in the world (Hertog et al., 2023), but the 2023 World Happiness Report ranked India 126 out of 137 included countries, suggesting it is one the unhappiest (Helliwell et al., 2023). In light of this observation, it is particularly important to understand the determinants of happiness in India. While the SHS has been psychometrically validated in Indian samples (Bengali-speaking, Pyne et al., 2020; Hindi-speaking, Singh & Husain, 2021), to our knowledge there is no validated translation of the SHS into Malayalam, a language predominantly spoken by over 32 million people in the southern Indian state of Kerala (Census Tables, 2011). Kerala is notable among the Indian states, as it has been lauded for high literacy rates, long life expectancy, and a strong public health system (Sathyan et al., 2023). However, few studies have explored happiness in Kerala specifically, despite its uniqueness in quality of life. In the present study, we aim to translate and validate the Malayalam version of the SHS, to support future research that explores both similarities and differences in subjective happiness across cultures.

## Methods

### Participants

The Kerala-Einstein Study (KES) is an ongoing study established in 2007 that recruits community-dwelling adults aged 60 and older (Sathyan et al., 2023). Exclusion criteria included previous physician diagnosed dementia, presence of acute and/or terminal illnesses, and/or presence of severe auditory or visual loss. Participants were recruited from urban (Baby Memorial Hospital, Parayancheri), rural (Kakkodi Community Health Center), and exurban (Meitra Hospital) sites in Kozhikode, a northern district of Kerala, India. As of December 2023, 656 participants (39% female, *M* age 68.9 ± 5.61 years) completed the Malayalam version of the Subjective Happiness Scale (SHS). All KES participants who completed the Malayalam SHS were included in the present study. This study was approved by the institutional review boards of the Albert Einstein College of Medicine and Baby Memorial Hospital. All participants provided written informed consent.

### Instruments

#### Subjective Happiness Scale

The Subjective Happiness Scale (SHS) is a brief measure of global subjective happiness with high internal consistency (Cronbach’s α ranging from 0.79–0.94) and good reliability (Lyubomirsky & Lepper, 1999). The scale includes four questions that ask participants if they consider themselves a happy person generally, as well as in relation to others: (1) “In general, I consider myself…” 1 = Not a very happy person to 7 = A very happy person, (2) “Compared with most of my peers, I consider myself…” 1 = Less happy to 7 = More happy, (3) “Some people are generally very happy. They enjoy life regardless of what is going on, getting the most out of everything. To what extent does this characterization describe you?” 1 = Not at all to 7 = A great deal, (4) “Some people are generally not very happy. Although they are not depressed, they never seem as happy as they might be. To what extent does this characterization describe you?” 1 = Not at all to 7 = A great deal. Item 4 is reverse-scored. Each question is rated on a 7-point scale and the final SHS score represents the mean of the four items, ranging from 1 to 7, with higher scores indicating greater happiness.

For the present study, the English version of the SHS was first translated to Malayalam language by a bilingual translator. This version was then back translated to English by an independent bilingual translator with similar qualifications as the original translator to ensure accuracy of translation. The multidisciplinary research team and local experts reviewed the final version regarding format, wording, grammatical structure of the sentences, similarity in meaning, and relevance. Any ambiguities and discrepancies regarding cultural meaning and colloquialisms or idioms in words and sentences of the instructions, the items, and the response format between the back-translation and the original instrument were discussed and resolved through consensus among the committee members to derive a final version of the instrument (available in Online Resource 1).

#### Geriatric Depression Scale (GDS-30)

The Geriatric Depression Scale (GDS-30) is a self-report questionnaire which measures depressive symptoms in older adults (Yesavage et al., 1982), and has been widely used in both clinical and research settings. The GDS-30 consists of 30 yes/no questions which ask participants to assess their mood over the past week, with scores ranging from 0 to 30. A score of 0–9 is considered “normal,” 10–19 is considered “mild depression,” and ≥ 20 is considered “severe depression”. The Malayalam version of the GDS-30 has been previously validated (Verma et al., 2021) and the short-form GDS-15 has previously been used in the KES sample (Nair et al., 2014; Wang et al., 2016).

#### Generalized Anxiety Disorder

The Generalized Anxiety Disorder (GAD-7) is a brief clinical assessment for the screening of anxiety (Spitzer et al., 2006). The GAD-7 asks participants how often, in the last two weeks, they have been bothered by seven different anxiety symptoms, with response options including “not at all,” “several days,” “more than half the days,” and “nearly every day”. Scores of 0, 1, 2, and 3 are assigned to each symptom, with scores ranging from 0 to 21. A score of 0–4 of indicates “no anxiety,” 5–9 indicates “mild anxiety,” 10–14 indicates “moderate anxiety,” and ≥ 15 indicate “severe anxiety”. The Malayalam version of the GAD-7 has been previously psychometrically validated (De Man et al., 2021).

### Procedure

The questionnaires (in Malayalam language) were completed as part of the larger KES battery (Sathyan et al., 2023). After receiving brief instructions about the Malayalam SHS questionnaire, participants were read each item by a research assistant, and asked to verbally respond using a 7-point scale. The experimenter provided clarification as necessary. A similar procedure was conducted for the GDS-30 and GAD-7 questionnaires.

### Data Analysis

Descriptive statistics (mean and standard deviation) were calculated for each item on the Malayalam SHS, as well for overall scores on the Malayalam SHS, GDS-30, and GAD-7. To assess internal consistency, we calculated Cronbach’s α and composite reliability (CR). We also calculated the correlations between each SHS item.

We analyzed the dimensionality of the Malayalam SHS. To do so, we first randomly split the sample into two halves. We conducted an exploratory factor analysis (EFA) on the first half of the sample and validated the suggested structure with a confirmatory factor analysis (CFA) on the second half of the sample. Given our overall sample size of 656, the half-samples (training sample *n* = 328 and test sample *n* = 328) were larger than the suggested sample size of 300 for factor analysis. The Kaiser–Meyer–Olkin measure of sampling adequacy (KMO; Kaiser, 1970) for the EFA training sample was 0.72 and Bartlett’s test of sphericity (Bartlett, 1951) was significant, *p* < 0.001, indicating acceptable sampling adequacy and sufficiently large correlations between the items. To validate our model, we used a CFA with maximum likelihood estimation and report several fit criteria. First, we report the Chi-square goodness of fit (χ^2^), which indicates a good fit when the associated *p*-value is non-significant, although a large sample size may influence this test (Hoe, 2008). We also report the comparative fit index (CFI), for which values greater than 0.90 indicate good fit (Kline, 2016). Finally, we report the root mean square error of approximation (RMSEA), for which values less than 0.05 indicate a good fit, and the standardized root mean residual (SRMR), for which values greater than 0.10 indicate a poor fit.

Evidence for convergent validity was assessed by calculating the Average Variance Extracted (AVE) of the Malayalam SHS, for which values greater than 0.5 indicate acceptable levels of validity, and examining the Pearson’s correlations between overall scores on the Malayalam SHS, GDS-30, and GAD-7 in the whole sample. We conducted independent-samples t-tests to assess the differences across sex (male and female) and age (60–74 years and 75 + years) groups. All statistical analysis was conducted using R (R Core Team, 2023).

## Results

The mean scores for each item on the Malayalam SHS as well as the mean overall scores for all scales are presented in Table 1. The mean overall score for the Malayalam SHS was 5.06 (± 1.18) out of a possible 7. The mean score of the individual items ranged from 4.92 (± 1.46) to 5.26 (± 1.52).

**Table 1:** Mean scores for all measures

	Total Sample *N* = 656	Females *N* = 257	Males *N* = 399	60–75 years *N* = 551	75 + years *N* = 105
	*M* (*SD*)	*M* (*SD*)	*M* (*SD*)	*M* (*SD*)	*M* (*SD*)
SHS Item 1	5.05 (1.31)	4.84 (1.41)	5.18 (1.22)	5.01 (1.31)	5.26 (1.30)
SHS Item 2	5.01 (1.31)	4.82 (1.45)	5.13 (1.21)	4.98 (1.30)	5.13 (1.36)
SHS Item 3	4.92 (1.46)	4.60 (1.60)	5.12 (1.33)	4.89 (1.48)	5.06 (1.39)
SHS Item 4	5.26 (1.52)	4.99 (1.63)	5.44 (1.41)	5.24 (1.51)	5.36 (1.54)
SHS Total	5.06 (1.18)	4.82 (1.27)	5.22 (1.09)	5.03 (1.19)	5.20 (1.23)
GDS-30	7.10 (5.72)	8.51 (6.18)	6.20 (5.21)	7.28 (5.89)	6.18 (4.65)
GAD-7	3.24 (4.48)	3.98 (5.07)	2.76 (3.99)	3.36 (4.62)	2.63 (3.61)

SHS: Subjective Happiness Scale, GDS-30: Geriatric Depression Scale, GAD-7: Generalized Anxiety Disorder

The Cronbach’s α for the Malayalam version of the SHS was 0.86 and the CR was 0.87 (Table 2). The items were moderately to strongly correlated with each other, presented in Table 3. Item 1 and Item 2 showed the highest correlation (*r* = 0.89, 95% CI 0.86–0.91) while Item 1 and Item 4 (the reverse-scored item) showed the lowest correlation (*r* = 0.43, 95% CI 0.36–0.53).

**Table 2:** Consistency and validity assessment of the Subjective Happiness Scale

	Total Sample *N* = 656	Females *N* = 257	Males *N* = 399	60–75 years *N* = 551	75 + years *N* = 105
Internal Consistency (Cronbach’s α, 95% CI)	.86 (.84, .88)	.86 (.82, .88)	.86 (.84, .88)	.87 (.85, .89)	.81 (.75, .87)
Composite Reliability	.86	.86	.87	.87	.84
Average Variance Extracted	.59	.58	.60	.60	.56
SHS-GDS-30 Correlation (*r*, 95% CI)	− .63 (− .68, − .58)	− .68 (− .73, − .62)	− .56 (− .62, − .48)	− .61 (− .67, − .55)	− .74 (− .82, − .64)
SHS-GAD-7 Correlation (r, 95% CI)	− .51 (− .57, − .46)	− .55 (− .64, − .44)	− .46 (− .53, − .37)	− .50 (− .56, − .44)	− .60 (− .72, − .45)

SHS: Subjective Happiness Scale, GDS-30: Geriatric Depression Scale, GAD-7: Generalized Anxiety Disorder, CI: Confidence Interval

**Table 3:** Correlations between individual items on the Subjective Happiness Scale

	SHS Item 1	SHS Item 2	SHS Item 3	SHS Item 4
SHS Item 1	1.0			
SHS Item 2	.89	1.0		
SHS Item 3	.64	.67	1.0	
SHS Item 4	.43	.44	.63	1.0

SHS: Subjective Happiness Scale

The results of our exploratory factor analysis indicated a single-factor solution. Kaiser’s eigenvalue-greater-than-one criteria suggested one factor, with one eigenvalue greater than 1 (2.55) and the inflexion in the scree plot also suggested one factor. The single-factor solution explained 64% of the variance. The factor loadings for items 1–4 were: 0.86, 0.89, 0.83, and 0.57, and the mean communality was 0.64. The confirmatory factor analysis of the single-factor structure produced the following fit criteria: χ^2^(2) = 80.5, *p* < 0.001; CFI = 0.91; RMSEA = 0.36, 90% CI 0.29–0.42; and SRMR = 0.095.

To assess convergent validity, we calculated the AVE of the Malayalam SHS, finding an AVE of 0.59 (Table 2). We also calculated the correlation between subjective happiness and negative mental health symptoms, presented in Table 2. We calculated the correlation between subjective happiness and depressive symptoms, finding a negative correlation between scores on the Malayalam SHS and the GDS-30 (*r* = – 0.63, 95% CI – 0.68– – 0.58). We also calculated the correlation between subjective happiness and anxiety symptoms, again finding a negative correlation between Malayalam SHS and GAD-7 scores (*r* = -0.51, 95% CI – 0.56–– 0.45).

Because of the imbalance of sex in our samples (39% female and 61% male), we assessed if there were sex differences in reported subjective happiness. Sex group differences are reported in Table 1. Overall, female participants reported lower mean levels of happiness (*M* 4.82 ± 1.27) compared to male participants (*M* 5.22 ± 1.09), *t*(654) = -4.30, *p* < 0.001. Internal consistency remained the same across sex (Cronbach’s α: female = 0.86, male = 0.86; CR: female = 0.86, male = 0.87). The AVE was 0.58 in female participants and 0.60 in male participants. The correlation between Malayalam SHS and GDS-30 scores was greater in female participants (*r* = – 0.68) compared to male participants (*r* = – 0.56), as was the correlation between Malayalam SHS and GAD-7 scores (*r*: female = -0.55, male = – 0.46). These results are presented in Table 2.

We also assessed if there were age-related differences in reported subjective happiness. We compared SHS scores between two age groups: 60–74 years (*N* = 551, *M* 5.03 ± 1.19) and 75 + years (*N* = 105, *M* 5.20 ± 1.12). Age group differences are reported in Table 1. We found that mean level of happiness was not significantly different between the groups, *t*(654) = − 1.36, *p* = 0.17. Internal consistency was similar across age groups (Cronbach’s α: 60–74 years = 0.87, 75 + years = 0.81; CR: 60–74 years = 0.87, 75 + years = 0.84). The AVE was 0.60 in the 60–74 age group and 0.56 in the 75 + age group. The correlation between Malayalam SHS and GDS-30 scores was lower in the 60–74 age group (*r* = − 0.61) compared to the 75 + age group (*r* = − 0.74), as was the correlation between Malayalam SHS and GAD-7 scores (*r*: 60–74 = − 0.50, 75 +  = − 0.60). These results are presented in Table 2.

## Discussion

In the present study, we validated a Malayalam version of a commonly used measure of subjective happiness. Our results indicate that our Malayalam translation of the Subjective Happiness Scale is a valid measure among community-dwelling older adults in India. Such measures are important to better understand happiness across and within cultures, and as India is linguistically diverse and now the most populous country in the world (Hertog et al., 2023), a Malayalam version of this scale is highly relevant in broadening our understanding of happiness and subjective well-being.

Our present results indicate that Indian participants using the Malayalam version of the SHS report similar levels of happiness (*M* = 5.06) as those using the Hindi version of the SHS (*M* = 5.13, Singh & Husain, 2021) as well as other languages, such as Spanish (*M* = 5.09, Extremera & Fernández-Berrocal, 2014). The mean happiness reported in our sample was lower than that of older adults in the original English SHS (*M* = 5.62, Lyubomirsky & Lepper, 1999), but slightly higher than other language samples such as Russian (*M* = 4.02, Lyubomirsky & Lepper, 1999), Malay (*M* = 4.27, Swami, 2008), and Greek (*M* = 4.86, Karakasidou et al., 2016). Variations in sample demographics or cultural norms surrounding happiness (Oishi et al., 2013) may explain these differences, but these results suggest that happiness is relatively consistent across groups, and demonstrate good conceptual equivalence across populations.

We also found the Malayalam version of the SHS to have a high level of internal consistency (Cronbach’s α = 0.86), which is in line with the originally reported value of the English SHS (Lyubomirsky & Lepper, 1999), as well as other languages (e.g., Shimai et al., 2004; Swami, 2008; Swami et al., 2009). We further validated this result by finding a composite reliability of 0.87. Additionally, the results of our factor analysis indicate that the Malayalam version of the SHS has a unidimensional structure, as was reported in the original SHS (Lyubomirsky & Lepper, 1999). Other studies which have investigated the dimensionality of the SHS, such as the Hindi (Singh & Husain, 2021), Tagalog (Swami et al., 2009), and German versions (Swami et al., 2009), have reported similar results. In our CFA of the single-factor solution, though two of our fit measures (CFI and SRMR) indicated a good fit, our χ^2^ and RMSEA values suggested a poor fit. However, χ^2^ is sensitive to large sample sizes (Hoe, 2008) and RMSEA may be interpreted cautiously with small degrees of freedom (Jovanović, 2014; Kenny et al., 2015), as in the present study. We further demonstrated that scores on the Malayalam version of the SHS were negatively correlated with scores on scales measuring symptoms of depression (*r* = − 0.63) and anxiety (*r* = − 0.51), as expected. Previous work has found similar correlations between SHS scores and depression and anxiety scores with the English (Lyubomirsky & Lepper, 1999), Spanish (Extremera & Fernández-Berrocal, 2014), and Italian (Iani et al., 2014) versions of the SHS.

Happiness has been reported to vary across age groups (Diener et al., 1999, 2018; Lyubomirsky & Lepper, 1999), as have symptoms of depression and anxiety (Teachman, 2006; Wolitzky-Taylor et al., 2010). While we found negative associations between happiness and depression symptoms and between happiness and anxiety symptoms in both age groups (60–74 years and 75 + years) in the present sample, the relationships were stronger in the older group. Additionally, the relationship between happiness and depressive symptoms was stronger in our sample (*r* = − 0.63) compared to some younger samples (e.g., *r* = − 0.52, Extremera & Fernández-Berrocal, 2014; *r* = − 0.49, Lyubomirsky & Lepper, 1999) but not others (*r* = − 0.61, Iani et al., 2014). The opposite pattern was seen for anxiety, with our older adult sample showing a slightly weaker relationship between happiness and anxiety symptoms (*r* = − 0.51), compared to younger samples (e.g., *r* = − 0.60, Extremera & Fernández-Berrocal, 2014; *r* = − 0.59, Iani et al., 2014). This finding may be due to different instruments used in each study, but future research should consider the relationships between happiness and mental health symptoms across age groups.

Our study has strengths, including a large sample of community-dwelling adults from a population which has been largely understudied in the field of happiness research. However, there are also several limitations of the present study. First, our study included only adults older than 60, because of the enrollment criteria of the Kerala Einstein Study, and our sample had a larger number of male participants compared to female participants. As we found a difference in reported happiness across sex, future research should expand the sample demographics to increase the generalizability of the results. Still, our findings are largely in line with previous research in college-age and other younger samples. Further, we were unable to compare our results to other measures of well-being, largely because of a present lack of Malayalam versions of other subjective well-being instruments. Though we did find evidence of the validity of the Malayalam version of the SHS using scales that measure depression and anxiety symptoms, more research is needed to confirm these findings using specific well-being measures.

The present study introduces several directions for future research exploring subjective well-being in diverse populations and a Malayalam version of the SHS scale is particularly useful. Malayalam is spoken by over 32 million people in the southern Indian state of Kerala, which itself is unique among Indian states, ranking the highest in the Human Development Index (Suryanarayana et al., 2016). Future directions could explore both cross-cultural determinants of happiness, as well as if and how people from Kerala demonstrate differences in happiness compared to those from other Indian states.

In conclusion, this study presents a Malayalam version of the Subjective Happiness Scale, the first to our knowledge, and reports that this version has high levels of internal consistency and convergent validity. Together, these results indicate that the Malayalam Subjective Happiness Scale is a useful tool to measure happiness in Malayalam-speaking populations. Expanding the availability of instruments in diverse languages is critical as future research continues to explore happiness and subjective well-being across and within cultures.

## Supplementary Information

Below is the link to the electronic supplementary material.

Supplementary file1 (PDF 110 kb)

## Data Availability

The Malayalam version of the Subjective Happiness Scale is available as online supplemental material. Data are available upon request and with completion of the data user agreement.
