# Supplementary material for: Translation and Validation of the Malayalam Version of the Subjective Happiness Scale
Source: Soc Indic Res. 2024 Oct 22;176(1):245–55. doi: 10.1007/s11205-024-03448-y (PMC11813988; doi:10.1007/s11205-024-03448-y)
Supplement: Supplementary file 1 — Supplementary file1 (PDF 110 kb) [file 11205_2024_3448_MOESM1_ESM.pdf]

Online Resource 1 Malayalam version of the Subjective Happiness Scale

ആത്മനിഷ്ഠമായി സന്തോഷം അളക്കുന്നതിനുള്ള സ്കെയിൽ

ഞാൻ നിങ്ങളോട് പ്രസ്താവനകളുടെയും/ചോദ്യങ്ങളുടെയും ഒരു ലിസ്റ്റ് വായിക്കും. ഓരോ പ്രസ്താവനകൾക്കും കൂടാതെ ചോദ്യങ്ങൾക്കും, നിങ്ങളെ വിവരിക്കുന്നതിൽ ഏറ്റവും അനുയോജ്യമെന്ന് നിങ്ങൾക്ക് തോന്നുന്ന സ്കെയിലിലെ പോയിന്റ് എന്നോട് പറയുക

1. പൊതുവേ, ഞാൻ എന്നെത്തന്നെപരിഗണിക്കുന്നത്:

   1 അത്ര സന്തോഷമുള്ള ഒരു വ്യക്തി ആയിട്ടല്ല
   2
   3
   4
   5
   6
   7 വളരെ സന്തോഷമുള്ള ഒരു വ്യക്തി ആയിട്ടാണ്

2. എന്റെ സമപ്രായക്കാരിൽ മിക്കവരുമായി താരതമ്യം ചെയ്യുമ്പോൾ; ഞാൻ എന്നെത്തന്നെപരിഗണിക്കുന്നത്:

   1 സന്തോഷം കുറവുള്ള വ്യക്തി ആയിട്ടാണ്
   2
   3
   4
   5
   6
   7 സന്തോഷം കൂടുതലുള്ള വ്യക്തി ആയിട്ടാണ്

3. ചിലർ പൊതുവെ വളരെ സന്തുഷ്ടരാണ്. എന്താണ് സംഭവിക്കുന്നതെന്ന് പരിഗണിക്കാതെ അവർ ജീവിതം ആസ്വദിക്കുന്നു, എല്ലാം പരമാവധി പ്രയോജനപ്പെടുത്തുന്നു. ഈ സ്വഭാവം നിങ്ങളെ എത്രത്തോളം വിവരിക്കുന്നു?

   1 ഒരിക്കലുമില്ല
   2
   3
   4
   5
   6
   7 എല്ലായിപ്പോഴും

4. ചിലർ പൊതുവെ അത്ര സന്തുഷ്ടരല്ല. അവർ വിഷാദരോഗികളല്ലെങ്കിലും, ഒരിക്കലും സന്തോഷവാൻമാരായി കാണപ്പെടുന്നില്ല. ഈ സ്വഭാവം നിങ്ങളെ എത്രത്തോളം വിവരിക്കുന്നു?

   1 ഒരിക്കലുമില്ല
   2
   3
   4
   5
   6
   7 എല്ലായിപ്പോഴും
